# Differential Roles of Hath1, MUC2 and P27Kip1 in Relation with Gamma-Secretase Inhibition in Human Colonic Carcinomas: A Translational Study

**DOI:** 10.1371/journal.pone.0055904

**Published:** 2013-02-11

**Authors:** Frédérique Souazé, Chantal Bou-Hanna, Christine Kandel, François Leclair, Julie Devallière, Béatrice Charreau, Stéphane Bézieau, Jean-François Mosnier, Christian L. Laboisse

**Affiliations:** 1 EA Biometadys, Université de Nantes, Nantes, France; 2 CHU Nantes, Service d’Anatomie et de Cytologie Pathologiques, Nantes, France; 3 UMR INSERM, U1064, Université de Nantes, Nantes, France; 4 CHU Nantes, Service de Génétique, Nantes, France; Columbia University Medical Center, United States of America

## Abstract

Hath1, a bHLH transcription factor negatively regulated by the γ-secretase-dependent Notch pathway, is required for intestinal secretory cell differentiation. Our aim was fourfold: 1) determine whether Hath1 is able to alter the phenotype of colon cancer cells that are committed to a differentiated phenotype, 2) determine whether the Hath1-dependent alteration of differentiation is coupled to a restriction of anchorage-dependent growth, 3) decipher the respective roles of three putative tumor suppressor genes Hath1, MUC2 and P27kip1 in this coupling and, 4) examine how our findings translate to primary tumors. Human colon carcinoma cell lines that differentiate along a mucin secreting (MUC2/MUC5AC) and/or enterocytic (DPPIV) lineages were maintained on inserts with or without a γ-secretase inhibitor (DBZ). Then the cells were detached and their ability to survive/proliferate in the absence of substratum was assessed. γ-secretase inhibition led to a Hath1-mediated preferential induction of MUC2 over MUC5AC, without DPPIV modification, in association with a decrease in anchorage-independent growth. While P27kip1 silencing relieved the cells from the Hath1-induced decrease of anchorage-independent growth, MUC2 silencing did not modify this parameter. Hath1 ectopic expression in the Hath1 negative enterocytic Caco2 cells led to a decreased anchorage-independent growth in a P27kip1-independent manner. In cultured primary human colon carcinomas, Hath1 was up-regulated in 7 out of 10 tumors upon DBZ treatment. Parallel MUC2 up-regulation occurred in 4 (4/7) and P27kip1 in only 2 (2/7) tumors. Interestingly, the response patterns of primary tumors to DBZ fitted with the hierarchical model of divergent signalling derived from our findings on cell lines.

## Introduction

Most colorectal cancers are of epithelial origin. Hallmarks of neoplastic epithelial cells include their relief (i) from the constraints of anchorage to a substratum for their survival/proliferation and (ii) from the so-called terminal differentiation. In fact, some colorectal carcinomas display an undifferentiated proliferative phenotype accounted for by a constitutively activated notch signalling [1]–[4]. The intracellular domain of the Notch receptor (NICD) is released upon γ-secretase activation, then enters the nucleus and maintains a negative control over Math1, whose human ortholog is Hath1, through the transcription repressor Hes [5]–[7]. Math1 is essential for adult intestinal secretory cell production, and in its absence cells destined to a secretory phenotype instead adopt an absorptive phenotype [8], [9]. Support for the control of cell fate by Hath1 in undifferentiated human colon cancer cells stems mainly from the use of Hath1 over-expression in the undifferentiated colon cancer cell line HT29 [10], [11]. Hath1 over-expression was shown to induce the expression of both MUC2 colonic mucins mRNAs and the cell cyle regulator P27Kip1 in association with a decreased survival/proliferation of cancer cells [11]. Interestingly Hath1, MUC2, P27kip1 are *per se* tumor suppressor candidates in the colon and are therefore candidates for coupling the arrest of proliferation to the differentiation of colon cancer cells [10], [12], [13]. However up to now, there has been no attempt to delineate their respective roles in restoring normal growth constraints upon Hath1 manipulation.

Undifferentiated carcinomas represent only a fraction of colonic cancers: a majority of carcinomas belong to the so-called moderately and well-differentiated categories of colon cancers [14]. Phenotypically, these carcinomas often display an abnormal differentiation that includes the acquisition of ectopic biomarkers [15] in addition to exhibiting either of two major lineages of intestinal differentiation, i.e. mucus-secreting or enterocytic. For example, mucus-secreting colorectal cancer cells often express MUC5AC gastric mucins together with MUC2 colonic mucins [16]–[19]. Colon cancer cells with an absorptive, i.e. enterocytic, differentiation display an apical brush-border endowed with the Dipeptidyl peptidase IV (DPPIV) small-intestinal hydrolase [20], [21]. Finally, as to whether the expression of an abnormal differentiation by colon cancer cells is mechanistically linked to their relaxation from anchorage-dependent survival/proliferation has remained unknown yet. At stake is the fact that restoring a normal differentiation of colon cancer cells could constrain them to normal growth conditions. This can be theoretically achieved by using γ-secretase inhibitors (GSIs), which block the generation of Notch intracellular domain and are therefore good candidates to modify the fate of cancer cells through Hath1 up-regulation. In this work we addressed the following 4 questions: Does γ-secretase inhibition modify the differentiation pattern of colonic cancer cells that are constitutively committed to differentiate into mucus-secreting (MUC2, MUC5AC) or enterocytic cells? Are these phenotypic changes coupled to a decreased anchorage-independent growth? If yes, what are the respective roles of Hath1, MUC2 and P27Kip1 in this process? Finally, how do our findings translate to human primary tumors?

To address these issues, we used several human colonic cancer cell lines differing in their differentiation commitment. HT29-Cl.27H is a clonal derivative of HT-29 cell line whose cells differentiate in post-confluent cultures into both mucus-secreting and enterocytic cells [22]. The Caco2 cell line differentiates at confluency into enterocytic cells [23]. Finally, HT29-Cl.16E cells differentiate at confluency into mucus-secreting cells [24]. All these cell lines exhibit an abnormal differentiation that is not terminal [25]. In other words, the differentiated cells maintain the potential to resume proliferation upon reseeding. In addition the cells exhibit the property of anchorage-independent growth.

In order to decipher the impact of Hath1 over-expression on cell differentiation, cells were first cultured with or without a γ-secretase inhibitor (DBZ) on an adhesion-permissive substrate,. Then, to address the issue of a possible connection between differentiation parameters and anchorage-dependent survival/proliferation, the differentiated cells were seeded in anchorage-independent conditions. A siRNA strategy was devised to delineate the signalling elements linking the parameters of differentiation to a restricted ability to survive/proliferate in anchorage-independent conditions. Finally, using a method of short term culture of primary human colonic carcinoma, we examined the response pattern of the tumor cells in terms of Hath1, MUC2 and P27Kip1 mRNAs to DBZ. This is to our knowledge the first attempt to decipher the hierarchy of 3 putative tumor suppressor genes in the coupling of the differentiation of human colon cancer cells to the restriction of anchorage-independent growth with a translation to human primary tumors.

## Materials and Methods

### Cell Lines and Culture on Filters

Cell lines: HT29-Cl.27H are committed to differentiate into mucus secreting cells and enterocytic cells upon reaching confluency in standard culture conditions (Dulbecco's Modified Eagle's Medium DMEM, 4.5 g/L glucose)/10% heat-inactivated FCS (Invitrogen, France). Caco2 cells are maintained in standard culture conditions (Dulbecco's Modified Eagle's Medium DMEM, 4.5 g/L glucose)/20% heat-inactivated FCS (Invitrogen, France). HT29-Cl.16E differentiates at confluency only into mucus secreting cells [24]. Finally, these cells were used to generate stable Flag-Sox9-inducible HT29-Cl.16E cells [26]. HT29-Cl.16E-Sox9 cells were cultured in standard conditions plus zeocyne 250 µg/ml and blasticidine 5 µg/ml. Flag-Sox9 was induced by addition of 2 µg/ml of doxycycline. All these cell lines displayed in culture the characteristics of differentiation that were described in the original papers [22], [23]. Culture on filters: Cells were seeded at high density, 1 million cells/well, on porous filters (12-well Transwell Clear, 0.45 µm porosity, Costar, France). γ-secretase inhibitor, DBZ (S)-2-(2-(3,5-difluorophenyl)acetamido)-N-((S)-5-methyl-6-oxo-6,7-dihydro-5H-dibenzo[b,d]azepin-7-yl) propanamide, Chemical Formula: C26H23F2N3O3 (syncom, The Netherlands) was used at 0.46 µM [27], [28]. We verified that at this concentration, a 21 day treatment had no cytotoxic effect on cells (data not shown). Control cells were treated with the vehicle DMSO (1∶10 000) and each agent was renewed every 2 days. Cyclin dependent kinase inhibitor, Flavopiridol (Sigma-Aldrich) was used at 10^−7^M.

The HT29-Cl.16E and HT29-Cl.27H epithelial cell lines were established in our lab [24] [22] as clonal derivatives of the HT29 cell line given by Dr Fogh [29]. Caco2 cells were obtained from the ATCC.

### Stable Transfection Experiments

Caco2 cells were grown in 6-well plates and transfected at a confluency of 80% with 1µg plasmid DNA (pCMV6-Myc tag-Hath1) (Origene), using 1.5 µl Nanojuice core transfection reagent and 1.5 µl Nanojuice transfection booster (Novagen). Polyclonal populations of stably transfected Caco2 cells were obtained by selection using 800 µg/ml of G418 (In vitrogen) for 2–3 weeks.

### Histology, Histochemistry and Immunohistochemistry of Cells Maintained on Filters

After 21 days of culture, filter-grown cells were fixed in AFA (Alcohol/Formalin/Acetic Acid) (Carlo Erba) during 30 min and dehydrated in increasing concentrations of ethanol. The filters were embedded in paraffin, sections were cut and stained with Haematoxylin/Eosin/Safran (HES) and alcian blue (pH 2.5), specific for acidic mucins. Immunohistochemistry was carried out using the avidin-peroxidase complex method after antigen retrieval in citrate buffer. Diaminobenzidine (DAB) was used as a chromogen. A light nuclear counterstaining was performed with haematoxylin. The following antibodies were used: MUC2 [mouse monoclonal antibody (1∶100; NCL-MUC2 Novocastra)], MUC5AC [45M1, mouse monoclonal antibodies (1∶1000; gift from Dr J. Bara, INSERM-UPMC U893, France)] [16], and DPPIV CD26 [goat polyclonal antibody (AF1180 1:100; R&D Systems)].

### Colony Formation Assay

A monodispersed cell suspension of HT29-Cl.27H or Caco2 cells was prepared from filter-grown cells by washing the filters with 1 mM EDTA in PBS followed by treating the cells with 0.25% trypsin in PBS-EDTA. Clonogenicity in soft agarose was determined by plating the cells in DMEM/10% FCS containing 0.35% agarose, over a layer of 0.5% agarose in the same culture medium [30]. Colonies, defined as aggregates of more than 10 cells, were counted using an inverted microscope, in each square of a plastic grid placed over the agarose layer.

### Anchorage-independent Culture

A monodispersed cell suspension, prepared as described above, was seeded in DMEM containing 10% heat-inactivated FCS at 10^6^ cells per well in pre-coated 12-well plates. Precoating was performed using polyhydroxyethylmethacrylate (polyHEMA, Sigma-Aldrich) dissolved at 10 mg/ml in ethanol. The ethanol was evaporated in a laminar flow hood. In these culture conditions, the cells are maintained in suspension.

### Infection by Adenoviral Vector Encoding Notch2ICD

HT29 Cl27H were cultured 70–90% confluency (250 000 cells in a 12 well plate) and infected with a moi of 30 or 40/cell for AdN2ICD, AdTrack-GFP and AdNull (Ad D1324) as previously described [31]. Transduction efficiency was analyzed 48h after infection through GFP detection by direct microscopy imaging and total RNA was prepared for quantitative analysis of transcripts by real-time RT-PCR (see below).

### siRNA Transfection

The siRNA on target plus (SMARTpool of four individual siRNAs duplex)(Dharmacon) targeting MUC2 (L-012643-01), Hath1 (L-008915-01), P27 (L-003472-00) and the scrambled siRNA control (D-001810-10) (a non-targeting siRNA pool siRNA-NT) were purchased from Dharmacon, Inc. siRNA transfections were performed using Dharmafect1 (Dharmacon). Total RNA extraction was performed 72 h after siRNA transfection.

### Cell Viability

Cell viability in suspension culture was quantified in hemacytometer chambers using trypan blue dye exclusion.

### Cytospin Preparations

The cells were suspended in PBS then centrifuged at 1200 rpm for 5 min (Cytospin3, ThermoShandon). The slides were fixed in cold acetone during 10 min and stored at –20°C until use. Immunocytochemistry was carried out on cytospin preparations using the avidin-peroxidase complex method. The following antibody was used : M30, specific for epithelial apoptotic cells [32] [mouse monoclonal antibody (1∶50; (12140322001), RocheDiagnostics)]. Negative control by omission of the primary antibody was performed in each experiment.

### Quantitative Analysis of Transcripts by Real-time RT-PCR

Total RNA was extracted using TRI REAGENT (Euromedex, France). Reverse transcription reaction was performed on 5 µg of total RNA as previously described [33]. Real time PCR analyses were performed using the following Taqman probes (Applied Biosystems) for MUC2 (HS00159374-m1); MUC5AC (HS00873638-m1); DPPIV (HS00175210-m1); HES1 (Hs00172878-m1). PCR products were quantified continuously with AB7000 (Applied Biosystems) according to the manufacturer’s instructions. Amplification for Hath1 and P27Kip1 was performed in a rotorgene using sybergreen for detection, primers sequence are for Hath1 forward: 5′-CCCCGGGAGCATCTTG-3′ reverse: 5′ GGGACCGAGGCGAAGTT-3′ and for P27Kip1 forward: 5′-GGGGCTCCGGCTAACTCTG-3′ reverse: 5′-GGCTTCTTGGGCGTCTGCTC-3′. The relative amounts of each transcript were normalized to human β-actin transcripts.

### Immunoblot Analysis

For total proteins extraction, cultured cells were homogenized in a stringent SDS-containing RIPA buffer as described previously [32]. Samples were loaded on 12% SDS-polyacrylamide gels (Bio-Rad). Proteins were electrotransferred onto nitrocellulose membranes (Bio-Rad). After blocking the membranes were probed with rabbit polyclonal antibodies directed to P27Kip1 (1∶500, Thermo Fisher Scientific, Fremont, CA 94538), c-myc (1∶500, clone 9E10, Origene) or β-actin (1∶30 000, clone AC-15, Sigma-Aldrich) followed by the corresponding horseradish peroxydase-conjugated antibody (1∶20,000; Jackson ImmunoResearch, West Grove, PA). The immunoreactive proteins were detected on films using an enhanced chemiluminescence substrate according to the manufacturer’s instructions (Roche Diagnostics).

### Primary Culture of Human Colon Carcinomas

Tumor samples were obtained following surgery from a series of consecutive patients with the informed consent of the patients and according to the guidelines of the French ethical law. The histopathological characteristics of each tumor, according to the WHO classification, are mentioned in Table 1 as well as nuclear β-catenin immunohistochemistry (monoclonal antibody clone CAT-5H10, 1/100, Invitrogen). Tumor samples were washed twice in antibiotics and antifungic solutions (penicillin, streptomycin, fungizone) in RPMI1640. Then, the fragments were cut into small pieces and incubated in collagenase at 37°C under agitation for 1 hour. The cell suspension consisting mainly of tumor cell clumps was centrifugated at 3000 rpm for 10 minutes and resuspended in complete medium (RPMI1640/FCS 10%, penicillin, streptomycin, fungizone) and debris and mucus were eliminated by filtering on gauze. The cell clumps were seeded in 6-well plates pre-coated with polyHEMA with 2 ml of complete medium and maintained for 24 hours with or without DBZ treatment (0.46 µM).

**Table 1:** Effect of γ-secretase inhibition on Hath1/MUC2/P27Kip1 expression in human colonic carcinomas in primary culture.

A Characteristics of the tumors				B Control culture(mRNA expression)			C DBZ response(fold change vs control)			
	Histopathological diagnosis according to the WHO classification	localization	Nuclear β-catenin staining(% positive nuclei)	Hath1	MUC2	P27	Hes1	Hath1	MUC2	P27
1	Well differentiated adenocarcinoma NOS*	Left colon	30%	+	−	++	0.4	2.7	u	u
2	Moderately differentiated adenocarcinoma NOS*	Left colon	30%	+	++	++	0.5	u	u	u
3	Moderately differentiated adenocarcinoma NOS* with a mucinous component	Right colon	< 5%	++	+	++	0.7	3	u	u
4	Mucinous adenocarcinoma	Rectum	< 5%	+	++	++	0.7	1.5	u	1.4
5	Poorly differentiated adenocarcinoma NOS*	Right colon	10%	+	+	++	0.7	2.5	1.5	1.4
6	Mucinous adenocarcinoma	Right colon	< 5%	–	++	++	0.7	u	u	u
7	Mucinous adenocarcinoma	Left colon	60%	+	++	+	0.5	2	1.6	u
8	Mucinous adenocarcinoma	Right colon	40%	+	++	++	0.6	2	1.8	u
9	Moderately differentiated adenocarcinoma NOS*	Rectum	60%	+	+	++	0.5	1.4	1.5	u
10	Poorly differentiated adenocarcinoma NOS*	Right colon	30%	+	−	++	0.7	u	u	u

* NOS: not otherwise specified (WHO classification), u = unresponsive tumor culture.

A: Characteristics of the primary tumors: histopathological diagnosis was according to the WHO classification of gastrointestinal tumors [42] and immunohistochemistry of nuclear β-catenin. B: Determination of Hath1, MUC2, P27Kip1 mRNA levels in control 24 h cultures was performed by quantitative RT-PCR. Results are normalized to β-actin gene expression. Arbitrary units were translated into a 3-tier scale; (−): absence of expression, (+): moderately expressed (1–10 arbitrary units), (++): highly to very highly expressed (20–100 arbitrary units). C: RT-PCR detection of Hes1, Hath1, MUC2 and P27Kip1 in 10 human colon carcinomas in primary culture with or without DBZ for 24 hours. Response to DBZ was expressed as the fold change versus control (Mean of 3 determinations).

### Sequencing

The ATOH1 open reading frame from 2 samples was amplified with PCR using AATAAGACGTTGCAGAAGAG and TCGCAGAGCAAAAATTAAAGGGTGC and Platinium Taq DNA polymerase and the GeneAmp PCR System 9700 (Applied Biosystems). The PCR products were purified and sequenced on a 3130 Genetic Analyser (Applied Biosystems).

### Statistical Analyses

Each experiment was performed at least in triplicate. Results were expressed as mean ± SEM. Statistical analyses were performed with GraphPad Prism version 4.0 (GraphPad software Inc.), using the Student-t test.

## Results

### Pharmacological Inhibition of γ-secretase Increased MUC2-secreting Goblet Cells: Morphological and Immunohistochemical Patterns of DBZ Treated Cells

**Figure 1:**
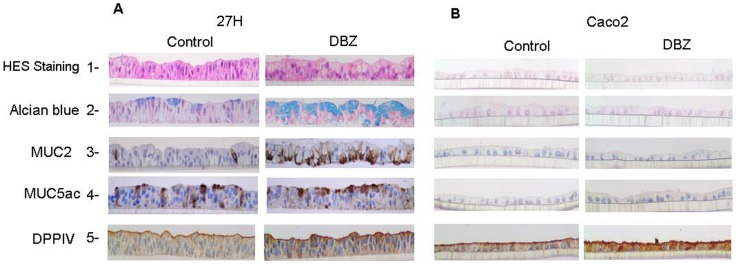
Morphological and immunohistochemical patterns of differentiation of filter-grown HT29-Cl.27H and Caco2 cells upon γ-secretase inhibition. HT29-Cl.27H (**A**) or Caco2 (**B**) cells were seeded at high density on filters and treated or not (control, DMSO 0.01%) with DBZ (0.46 µM) for 21 days (see Material and Methods). Then the filters were fixed, embedded in paraffin and sectioned perpendicularly to the culture plane. The slides were stained with HES and Alcian Blue. MUC2, MUC5AC, and DPPIV expression were detected by immunohistochemistry as described in material and methods.

**Figure 2:**
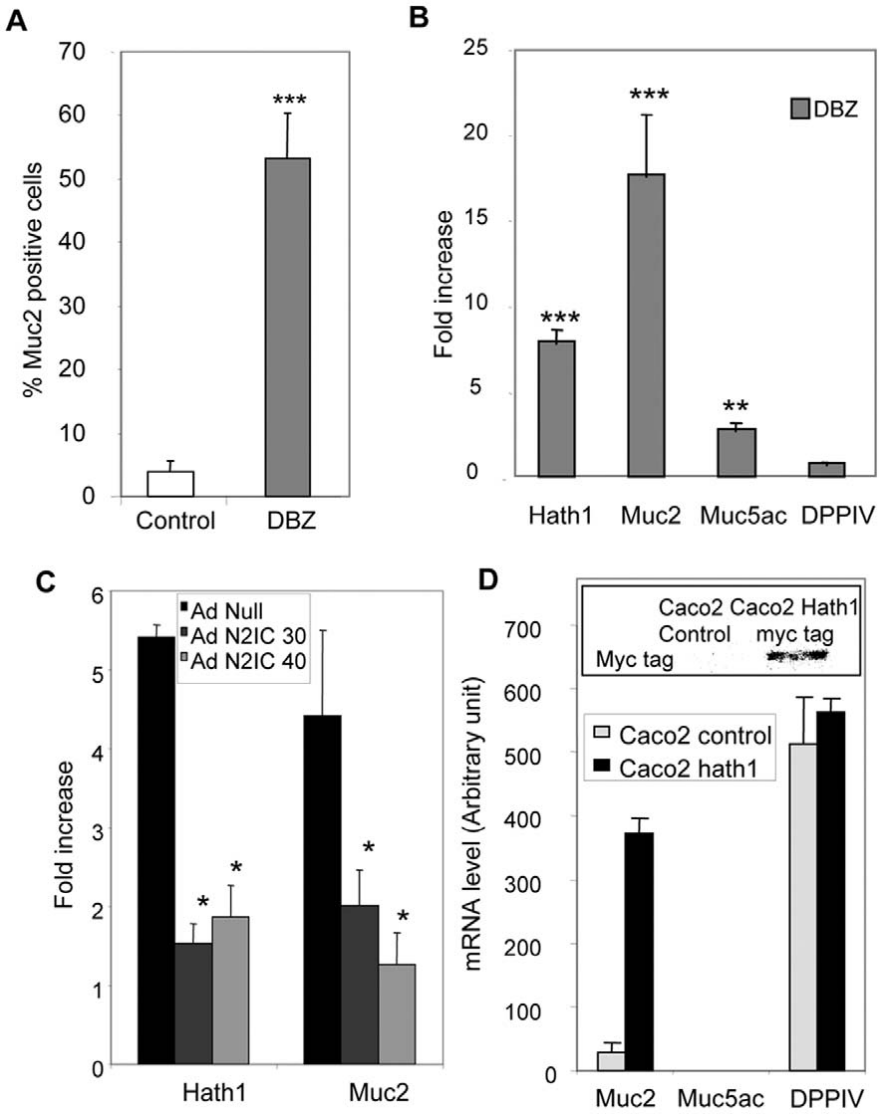
Effect of γ-secretase inhibition on the percentage of MUC2 positive cells and the expression of differentiation-associated genes. **A**- Percentage of MUC2 positive cells in control and DBZ-treated cells maintained on filters was determined by immunohistochemistry as described in material and methods. Mean ± SEM of 3 filters after counting at least 200 cells/filter (***, p<0.001 DBZ vs control). **B**- RT-PCR detection of Hath1, MUC2, MUC5AC, DPPIV mRNA in HT29-Cl.27H filter cultures treated with DBZ for 21 days. The results of real-time quantitative PCR are expressed relative to the expression level of control cultures after normalization to actin gene expression. Mean relative expression to the control DMSO; Mean ± SEM of 4 experiments; ***, P<0.001; **, P<0.01 (DBZ vs control). **C**- RT-PCR detection of Hath1 and MUC2 mRNA in DBZ-treated HT29-Cl.27H cells infected by AdN2ICD Adenovirus (30 or 40 moi/cell); Mean ± SEM of 3 experiments; *, P<0.05. **D**- RT-PCR detection of MUC2, MUC5AC and DPP-IV mRNA in polyclonal populations of Caco2 cells stably transfected by Hath1 expression vector. (For comparison purpose the Cts for MUC2 PCR are: Caco2-cmv Ct = 32; Caco2-Hath1 Ct = 26.5 and Ht29-Cl.27H Ct = 26). Insert: c-myc tag was detected by immunoblot on polyclonal populations of Caco2 cells stably transfected by empty vector or Hath1 expression vector.

HT29-Cl.27H cells maintained for 21 days on porous inserts formed polarized monolayers (Figure 1A, lane 1). Alcian blue, a stain for acidic mucin that is normally restricted to the intestinal goblet cells, stained some epithelial cells (Figure 1A, lane 2). Accordingly, immunohistochemistry showed that only few mucin-secreting cells expressed MUC2. The staining was restricted to the perinuclear region corresponding to the cis-Golgi since the antibody was specific for the peptidic epitope of human MUC2 (Figure 1A, lane 3). Numerous cells were stained by a mix of monoclonal antibodies specific for both the peptidic and carbohydrate chains of MUC5AC mucins that are normally expressed by the gastric mucosa (Figure 1A, lane 4). In addition, enterocytic cells were present, with an apical brush border stained with anti-DPPIV antibody (Figure 1A, lane 5). A 21-day treatment with DBZ of filter-grown cells was not cytotoxic as shown on HES staining (Figure 1A, lane 1). Interestingly, DBZ treatment led to a dramatic increase in alcian blue positive goblet cells (Figure 1A, lane 2). This was paralleled by a 6 to 9 fold increase in MUC2 positive epithelial cells (Figure 1A, lane 3 and Figure 2A). Finally, the staining patterns of MUC5AC and DPPIV did not change (Figure 1, lanes 4–5). This finding suggested that γ-secretase inhibition had no effect on the enterocytic differentiation.

To extend our study of the effect of γ-secretase inhibition on enterocytic differentiation, we used the Caco2 cell line as a model. As shown in figure 1B, filter-grown Caco2 cells dispayed a homogeneous differentiation into enterocytic cells as shown by DPPIV staining. In addition, after a 21-day treatment with DBZ there was no characteristic of differentiation into mucin producing cells.

### Signalling Pathway Underlying the DBZ-induced Differentiation

As shown in figure 2B, γ-secretase inhibition led to a concomitant increase in Hath1 (6–7 fold) and MUC2 (16–17 fold) mRNA expression and a slight increase in MUC5AC mRNA (3–4 fold) without significant alteration of DPPIV mRNA levels (Figure 2 B). Interestingly, these DBZ effects were accounted for by a blockade of the Notch signalling pathway as they were reversed by introducing a N2IC adenovirus (Figure 2C).

In Caco2 cells, consistent with the absence of morphological changes and differentiation pattern both in control and DBZ treated cells, there was no detectable level of Hath1 encoding mRNA. DPP-IV mRNA level remained unchanged upon DBZ treatment (data not shown). Interestingly, the ectopic expression of Hath1 in Caco2 cells (insert, figure 2D) led to a dramatic induction of MUC2 mRNA without effect on MUC5AC and without modification of DPP IV mRNA level (Figure 2D).

In conclusion, our results show that γ-secretase inhibition led to an increase of MUC2 positive cells.

### MUC2 Over-expression Upon γ-secretase Inhibition is Associated with Reduced Anchorage-independent Growth

**Figure 3:**
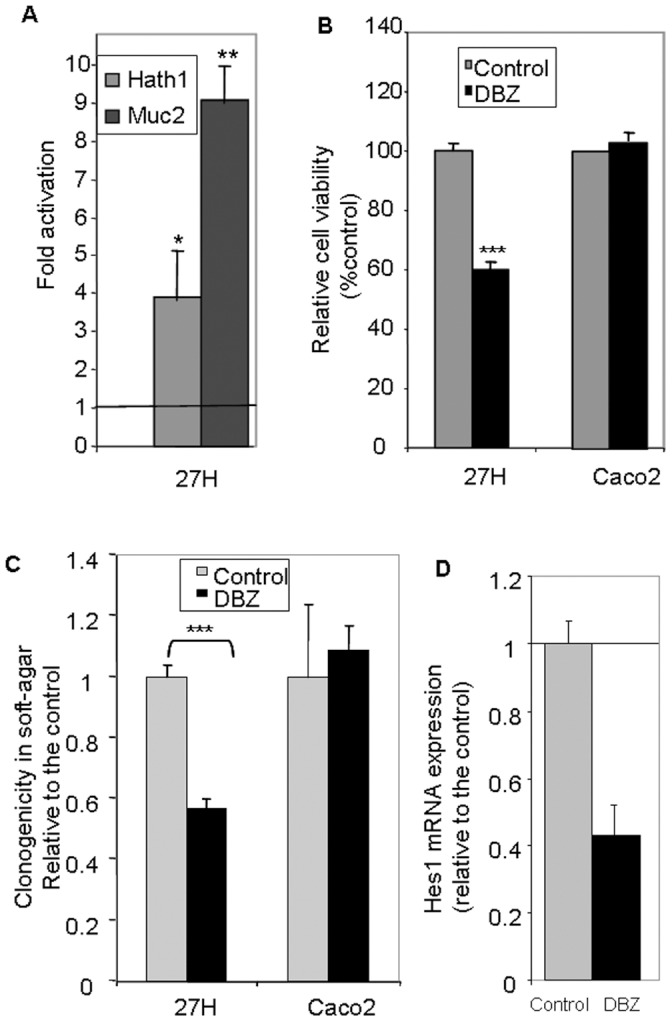
Effect of γ-secretase inhibition on cellular viability in anchorage-independent culture conditions in relation with Hath1 and MUC2 expression. **A and B-** Filter-grown HT29-Cl.27H or Caco2 cells, treated or not (control) with DBZ for 21 days, were resuspended and plated on polyHEMA-coated wells (nonadherent conditions) for 72 hours culture in 10% FCS containing medium without DBZ. **A**- RT-PCR detection of Hath1 and MUC2 mRNA: mean expression relative to control DMSO after normalization to actin gene expression; Mean ± SEM of 3 experiments; *, P<0.05; **, p<0.01. **B**- Relative cell viability versus control determined by trypan blue dye exclusion cell count. **C**- Filter-grown HT29-Cl.27H or Caco2 cells, pre-treated or not (control) with DBZ for 21 days were detached and seeded at 10, 000 cells per 60 mm dish in 0.35% agarose. The cells were maintained in culture for additional 15 days without supplementation of DBZ. Clusters of minimum 10 cells were counted. Mean relative expression to the control DMSO; Mean ± SEM of 3 experiments (3–4 dishes per experiment); ***, p<0.001. **D**- RT-PCR detection of Hes1 in Caco2 cells, treated or not (control) with DBZ for 21 days on filters: mean expression relative to control DMSO after normalization to actin gene expression; Mean ± SEM of 3 experiments.

To examine the impact of differentiation on the anchorage-independent growth, HT29-Cl27H cells were first induced to differentiate by DBZ and then the cells were detached and maintained without DBZ in suspension culture in polyhema coated wells. After 72 hours culture, MUC2 and Hath1 mRNA levels remained elevated in DBZ-pretreated cells (Figure 3A). Parallel to this maintenance of differentiation, we observed a 40% decrease in anchorage independent cell growth (Figure 3B). This effect resulted from a reduced proliferation and a 2 fold increase in cell apoptosis determined by the percentage of M30 positive cells (DBZ: 4.5% ±0.29 versus control: 1.9% ±0.38, ** p<0.01). DBZ-pretreated cells displayed a 50% decrease in soft agarose clonogenicity (Figure 3C). Interestingly, DBZ-pretreated Caco2 cells showed no modification of anchorage independent growth in suspension culture or in a soft agarose clonogenicity assay (Figure 3B and C). Although in Caco2 cells DBZ treatment did not alter Hath1 mRNA level, it was able to act upstream of Hath1 by decreasing Hes1 mRNA expression (Figure 3D).

### Hath1 Controls both Cellular Growth and MUC2 Expression in Anchorage-independent Culture Conditions

**Figure 4:**
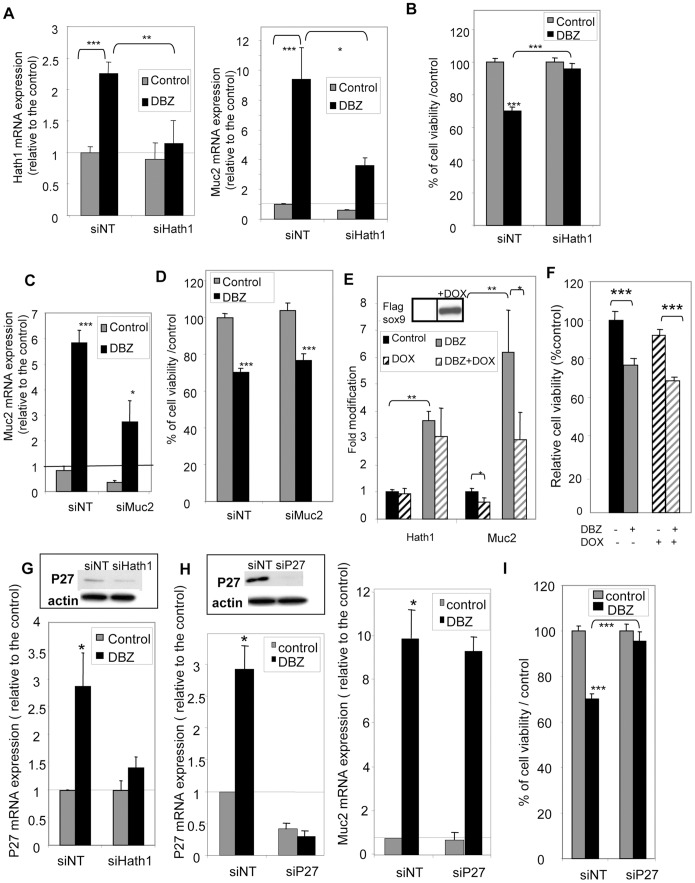
Effect of Hath1, MUC2 or P27Kip1 gene silencing on anchorage independent-growth. **A,B, C,D,G,H,I-**Filter-grown HT29-Cl.27H, treated or not (control) with DBZ for 21 days, were resuspended and plated on polyHEMA-coated wells (nonadherent conditions) for 72 hours culture in the presence of the indicated siRNA target smart pool (NT, non target; Hath1; MUC2 or P27Kip1). **A**- RT-PCR detection of Hath1 (left) and Muc-2 (right) mRNA in HT29-Cl.27H cells transfected by siRNA (NT, or Hath1): mean expression relative to siNT DMSO after normalization to actin gene expression; Mean ± SEM of 3 experiments; *, P<0.05; **, p<0.01; ***, p<0.001. **B**- Relative cell viability determined by Trypan blue dye exclusion cell count. Mean ± SEM of 3 experiments; ***, p<0.001. **C**- RT-PCR detection of MUC2 mRNA in HT29-Cl.27H cells transfected by siRNA (NT and MUC2): mean expression relative to siNT DMSO after normalization to actin gene expression; Mean ± SEM of 3 experiments; *, P<0.05; ***, p<0.001. **D**- Relative cell viability determined by Trypan blue dye exclusion cell count. Mean ± SEM of 3 experiments; ***, p<0.001. **E-** Filter-grown HT29-Cl.16E sox9 cells, treated or not (control) with DBZ for 21 days, were resuspended and plated on polyHEMA-coated wells (nonadherent conditions) for 72 hours culture in 10% FCS containing medium with or without addition of doxycycline. RT-PCR detection of Hath1 and MUC2 mRNA: relative expression to control DMSO after normalization to actin gene expression; Mean ± SEM of 3 experiments; **, P<0.01; *, P<0.05. Insert: Flag-Sox9 was detected by immunoblot on cellular extracts after 72-hour doxycycline induction. **F**- Relative cell viability determined by Trypan blue dye exclusion cell count (in the same culture conditions as Fig. 4E). Mean ± SEM of 3 experiments; ***, p<0.001. **G**- **and H**- RT-PCR detection of P27Kip1 mRNA (G and H left) and MUC2 mRNA (H right) in HT29-Cl.27H cells transfected by siRNA (G -NT and Hath1, H- NT and P27): mean expression relative to siNT DMSO after normalization to actin gene expression; Mean ± SEM of 3 experiments; ***, p<0.001. Insert: P27Kip1 and β-actin were detected by immunoblot on DBZ pre-treated cells transfected by siRNA (G -NT and Hath1, H left- NT and P27). **I**- Relative cell viability determined by Trypan blue dye exclusion cell count of HT29-Cl.27H cells transfected by siRNA NT or P27; Mean ± SEM of 3 experiments; ***, p<0.001.

To examine whether there was a common control of anchorage independent cellular growth and MUC2 expression upon γ-secretase inhibition, we performed Hath1 silencing by RNA interference in suspension culture. As shown in figure 4, transiently transfected Hath1 siRNA was able to significantly reverse the effect of DBZ on Hath1 expression (Figure 4A, Left) and concomitantly reduced MUC2 expression (Figure 4A, Right). In parallel, Hath1 siRNA reversed the inhibitory effect of DBZ on anchorage-independent growth (Figure 4B).

### MUC2 Down-regulation does not Interfere with DBZ-induced Reduction of Anchorage-Independent Growth

We used two approaches to inhibit MUC2 expression in DBZ-pretreated cells maintained in anchorage independent conditions. As shown in figure 4C, siRNA induced a 50% reduction in MUC2 mRNA expression in HT29-Cl.27H without modification of the reduced viability (Figure 4D) upon γ-secretase inhibition. Another approach consisted in using HT29-Cl.16e-Sox-9 cells, in which MUC2 expression can be down-regulated through a doxycycline-inducible Sox-9 [26]. We first verified in this cell line that a 21-day DBZ treatment led to a significant increase in MUC2 mRNA levels paralleled by an increase in Hath1 mRNA level (figure S1).

As shown in the insert of figure 4E, a 72-hour suspension culture of the cells in the presence of doxycycline led to a dramatic increase in the Flag-sox9 expression. Sox9 induction (Dox) was paralleled by a significant MUC2 mRNA decrease in both control and DBZ-pretreated suspension cultures without altering Hath1 mRNA expression (Figure 4E). Finally, Sox9 over-expression did not modify the anchorage independent growth in DBZ-pretreated cells (Figure 4F).

In conclusion, reduction in anchorage independent cellular growth upon γ-secretase inhibition was dependent of Hath1 expression independently of its effect on MUC2.

### Hath1-dependent P27Kip1 Expression is Responsible for the Reduction of Cellular Growth in Anchorage-independent Culture Conditions

P27Kip1 a cyclin-dependent kinase inhibitor, known to be regulated by Hes1 and/or Hath1 [11], [34], was up-regulated at the mRNA level in DBZ-treated HT29-Cl.27H. This P27Kip1 over-expression was maintained upon DBZ removal in suspension culture (Figure 4G). Hes1 mRNA down-regulation by DBZ treatment was not modified by siHath1 or siP27 (Figure S2). Interestingly, Hath1 silencing in anchorage-independent conditions led to a decrease of P27Kip1 mRNA level (Figure 4G). Finally, P27Kip1 silencing via siRNA (Figure 4H, left) reversed the effect of γ-secretase inhibition on anchorage-independent growth (Figure 4I) without any modification in the DBZ-induced MUC2 mRNA level (Figure 4H, right). Consistent with the known inhibition of cyclin-dependent kinase by P27Kip1, we found that flavopiridol (10^−7^M) an inhibitor of cyclin-dependent kinase, was able to reduce the anchorage independent growth of HT29-Cl.27H maintained on polyhema (flavopiridol: 115 200±1599 versus Control: 399 500±17 953 viable cells, **p<0.001) whereas it had no effect on the differentiation of the cells maintained on porous filters (data not shown).

### P27Kip1 Status of Caco2 Cells

Surprisingly, P27Kip1 constitutive expression in Caco2 cells was very high, therefore suggesting the existence of a Hath1-independent control of P27Kip1 in this cell line.

**Figure 5:**
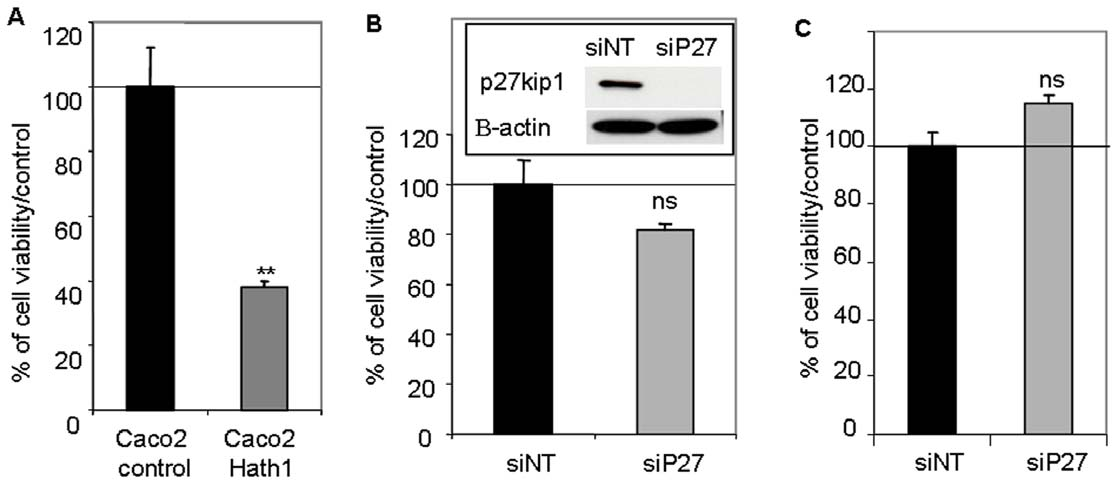
Effect of Hath1 ectopic expression and P27Kip1 silencing on anchorage-independent cell growth of Caco2 cells. A- Polyclonal populations of Caco2 cells stably transfected by empty vector (Caco2 control) or Hath1 expression vector (Caco2 Hath1) were plated on polyHEMA-coated wells (nonadherent conditions) for 72 hours culture in 10% FCS containing medium. Relative cell viability determined by Trypan blue dye exclusion cell count. Mean ± SEM; **, p<0.01. B-C- Caco2 cells (B) or Hath1 over-expressing Caco2 cells (C) plated on polyHEMA-coated wells (nonadherent conditions) for 72 hours culture with siRNA target smart pool (NT or P27). Relative cell viability determined by Trypan blue dye exclusion cell count. (B) Insert: P27Kip1 and β-actin were detected by immunoblot on cells transfected by siRNA.

However, Hath1 ectopic expression in Caco2 cells resulted in a reduced anchorage-independent growth of the cells (Figure 5A). Remarkably, P27Kip1 silencing by siRNA did not modify the anchorage-independent growth of parental Caco2 cells or Hath1-overexpressing Caco2 cells (Figure 5B and 5C). Thus the forced expression of Hath1 via genetic manipulation of a Hath1 negative colonic cancer cell line, resulting in very high Hath1 expression, negatively regulates cell proliferation in a manner that is clearly independent from that depending on the pharmacological inhibition of γ-secretase in Hath1 positive colonic cancer cells.

### Translation to Human Primary Colonic Cancer Cells (Table 1)

In order to examine whether these findings translate to carcinoma cells derived from cancer patients, we maintained primary colonic carcinomas in short-term culture so they are close to the in vivo situation. On the 20 successive tumors set into culture only 10 were amenable to RT-PCR analysis due to a poor viability even in short term cultures. In control cultures, only one lacked Hath1 expression (Table 1). Interestingly, there was a good correlation between the mRNA expression level of MUC2 in control cultures and the existence of the mucinous component of the parent tumor. DBZ treatment led to a down-regulation of Hes1 mRNA in all cases, an up-regulation of Hath1 mRNA in 7/10 cases and to a concomitant up-regulation of Hath1, MUC2 and P27Kip1 in only one case (case 5) (Table1, C). Interestingly, an up-regulation of MUC2 or P27Kip1 was always associated with Hath1 augmentation. Finally, MUC2 and P27Kip1 could be up-regulated separately by DBZ (case 4, 7, 8 and 9). As six out of ten tumors were unresponsive to DBZ in terms of MUC2 expression we examined the β-catenin status of the primary tumors as it was shown that hyperactivation of β-catenin signalling overrides the forced differentiation induced by Notch inhibition. Table 1 shows no correlation between the β-catenin status of the primary tumors, as determined by the percentage of β-catenin positive nuclei, and the extent of the response to DBZ treatment. Finally, Hath1 sequencing performed in 2 primary tumors (case 1 and 3) did not show any mutation of Hath1 gene that could explain the uncoupling of Hath1 up-regulation from MUC2 and P27Kip1 regulation (data not shown).

## Discussion

Several lines of investigation indicate that in intestinal progenitors, Math1 is the critical transcription factor in regulating secretory cell differentiation. Ablation of Math1 in mice was shown to change the cell fate, i.e. enterocytic lineage over secretory lineage [9]. Conversely, in mice, γ-secretase inhibition led to an augmentation of Math1 and a massive conversion of proliferative crypt cells into goblet cells [35]. Finally, intestinal stem cells lacking Math1 are refractory to γ-secretase inhibitors [36].

To address the issue of the cell fate change by γ-secretase inhibition in colonic cancer cells, we used several cell lines models as well as human primary carcinoma cells. The human colonic clonal cancer cell line, HT29-Cl.27H, is constitutively committed to differentiate at confluency along the two main intestinal lineages, absorptive/enterocytic and mucin secretory [22]. It is therefore a unique model to study whether γ-secretase inhibition is able to influence the binary decision between absorptive and secretory cell fate in colon cancer cells. Caco2 is committed to differentiate into the absorptive/enterocytic lineage [21] and HT29-Cl.16E is committed to the mucin secretory lineage [24].

Here we show that, by analogy with its effect on the mouse intestinal crypt, γ-secretase inhibition converted the colon cancer cells into differentiated cells with a goblet cell phenotype. Interestingly, the enterocytic differentiation remained unchanged upon γ-secretase inhibition in both HT29-Cl.27H and Caco2. Our experiments with Caco2 cells that are deficient in Hath1 or that are genetically engineered to over-express Hath1 confirm that the enterocytic differentiation of cancer cells is independent from the Hath1 pathway. Finally, as expected from the studies of normal cells, cancer cells lacking constitutive expression of Hath1, e.g. Caco2 cells, are refractory to γ-secretase inhibition.

Up to now the impact of γ-secretase inhibitors on the differentiation of colonic cancer cells has been evaluated only on the basis of MUC2 gene expression [1], [37], without taking into account the fact that colonic cancer cells ectopically express the MUC5AC gastric mucin [18]. In this context, it was important to examine the possibility that γ-secretase inhibition could differentially affect MUC2 and MUC5AC expression by cancer cells. From our experiments, it was clear that γ-secretase inhibition led to a preferential expression of MUC2. This change was due to a conversion of MUC2 negative cells into MUC2 positive cells. Finally MUC5AC was only weakly up-regulated by DBZ treatment. This differential effect of γ-secretase inhibition strongly suggests that Hath1 over-expression is able to restore a normal mucus secreting phenotype in colonic cancer cells.

Our strategy for evaluating survival/proliferation of the colonic cancer cells was based on the hypothesis that the differentiation induced by a γ-secretase inhibitor would remain stable upon removal of the agent and reseeding in anchorage-independent conditions. In fact, removal of the γ-secretase inhibitor did not lead to a reversal of differentiation and is in line with the concept derived from T-Cell acute lymphoblastic leukaemia cells that γ-secretase inhibition leads to a cell cycle exit upon removal of the inhibitor [38]. This was accompanied by a marked reduction of cell clonogenicity in soft agarose as well as a diminution of survival/proliferation in suspension culture and increased apoptotic index. All these elements indicate a partial recovery upon γ-secretase inhibition of normal constraints for survival/proliferation. As expected Caco2 cells lacking Hath1 was refractory to γ-secretase inhibition in relation with anchorage independence growth assays.

As the acquisition of stable characteristics of differentiation involved the over-expression of two putative tumor suppressor genes i.e. Hath1 [10] and MUC2 [12], it was important to decipher their respective role in the restoration of normal constraints for growth. Our strategy was to examine the effects of silencing either Hath1 or MUC2 in differentiated colonic cells maintained in anchorage-independent culture conditions. Basically, we used a MUC2 siRNA approach and a complementary approach based on MUC2 repression by Sox-9 [26]. Both approaches led to a significant reduction of MUC2 expression without altering the reduction of growth in anchorage-independent culture. Interestingly, in the same anchorage-independent culture conditions the Hath1 silencing led to a reduction of MUC2 expression and an increase in cell growth. This led to the conclusion that Hath1 was responsible for both the reduction of anchorage-independent growth and the goblet cell differentiation.

At that time it was therefore important to determine the Hath1-dependent pathway underlying the reduction of growth in anchorage independence. Was it included or not in the pathway regulating MUC2? Experiments of Notch ablation in mice showed that loss of intestinal crypt progenitor cells was accompanied by the up-regulation of two cyclin-dependent kinase (CDK) inhibitors, P27Kip1 and p57Kip2 [39]. In our experiments, P27kip1 mRNA was up-regulated upon γ-secretase inhibition (this article) but not p57Kip2 mRNA (Souazé et al, unpublished data). P27Kip1 was shown to be regulated by Hes1 acting directly on the promoter region [34]. In addition Leow et al found in colon cancer cells a P27Kip1 regulation by Hath1 [11]. Here we show using a siRNA strategy that in our specific conditions, i.e. DBZ treatment, P27Kip1 up-regulation is under the control of Hath1. Interestingly, P27Kip1 silencing did not reverse MUC2 expression of the differentiated cells. However, it was able to prevent the Hath1-induced reduction in anchorage-independent growth. Consequently it is possible that colonic cancer cells over-expressing MUC2 without the concomitant expression of P27Kip1 are relieved from the constraints of terminal differentiation and anchorage-dependent growth. Interestingly, Caco2 cells that are constitutively committed to differentiate only into enterocytic cells lack Hath1 and constitutively express high level of P27Kip1. In line with previous findings [40], P27Kip1 is not involved in the regulation of proliferation in this cell line. Thus the reduced anchorage-independent growth induced by ectopic expression of Hath1 is independent from P27Kip1 in this cell line.

**Figure 6:**
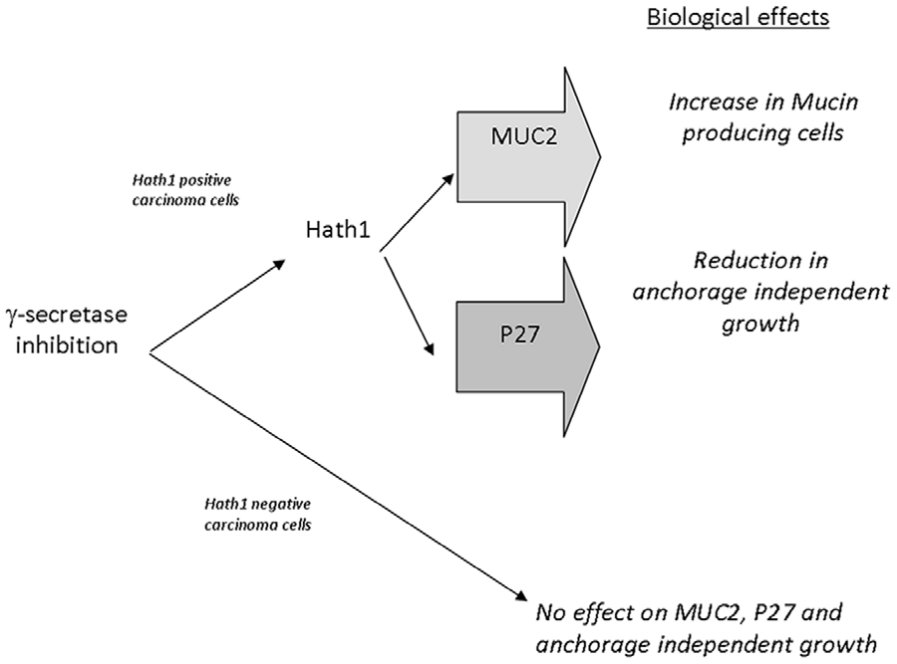
Proposed divergent signalling for gamma-secretase-dependent pathways of differentiation and anchorage-independent growth in colon cancer cells. Biological effects induced by γ-secretase inhibition in colonic cancer cells were dependent on Hath1 expression. In Hath1 non-expressing cells, γ-secretase inhibition had no effect on MUC2, P27Kip1 and anchorage independent growth. In Hath1 positive cells, γ-secretase inhibition led to an increase of Hath1 expression leading to two separate effects on 1- increase in mucin producing cells with MUC2 expression and 2- reduction in anchorage independent growth mediated by the activation of the cyclin dependent kinase inhibitor P27Kip1.

Whether the primary colonic cancer cells are able to respond to γ-secretase inhibition is an important issue. Due to the known difficulties for maintaining primary colon carcinomas in culture, we optimized a short term culture method. Interestingly, while all tumors displayed a Hes1 mRNA down-regulation upon DBZ treatment, only a minor fraction of the primary tumors displayed a “full” response to DBZ, i.e. an up-regulation of Hath1, MUC2 and P27Kip1. Interestingly Peignon et al [41] recently reported that the hyperactivation of β-catenin signalling overrides the forced differentiation induced by Notch inhibition. However in our experiments we did not find a correlation between β-catenin activation in the primary tumors, as shown by counting the percentage of β-catenin positive nuclei, and the level of Hath1, MUC2 and P27Kip1 expression in DBZ-treated tumor cells. In addition, we did not find any Hath1 mutation that could explain in 2 cases the uncoupling of Hath1 over-expression from MUC2 and P27Kip1 regulation. The response to DBZ fits with our proposed scheme of signalling pathways (Figure 6), (i) MUC2 and-or P27Kip1 augmentation appears to be associated with Hath1 up-regulation, and (ii) P27Kip1 and MUC2 can be separately up-regulated, a finding that is in line with a Hath1-dependent divergent signalling. In addition, P27Kip1 up-regulation upon DBZ treatment occurred only in a minor proportion of primary colon carcinomas. It comes then that a screening strategy based on primary culture of tumor cells is advisable for selecting patients that could response to γ-secretase inhibitors.

## Supporting Information

Figure S1 **Effect of γ-secretase inhibition on differentiation parameters of HT29-Cl.16E-Sox9 cells.** RT-PCR detection of Hath1, MUC2, MUC5AC mRNA in HT29-Cl.16E-Sox9 filter cultures treated with DBZ for 21 days. The results of real-time quantitative PCR are expressed relative to the expression level of control cultures after normalization to β-actin gene expression. Mean expression relative to control DMSO; Mean ± SE of 4 experiments; ***, P<0.001; **, P<0.01; *, P<0.05 (DBZ vs control).
(TIF)

Figure S2 **Effect of γ-secretase inhibition on Hes1 mRNA expression.** Filter-grown HT29-Cl.27H, treated or not (control) with DBZ for 21 days, were resuspended and plated on polyHEMA-coated wells (nonadherent conditions) for 72 hours culture in the presence of the indicated siRNA target smart pool (NT, non target; Hath1 or P27Kip1). RT-PCR detection of Hes1 mRNA mean expression relative to siNT DMSO after normalization to actin gene expression; Mean ± SEM of 3 experiments.
(TIF)
