# Supplementary figures and images for: Differential Roles of Hath1, MUC2 and P27Kip1 in Relation with Gamma-Secretase Inhibition in Human Colonic Carcinomas: A Translational Study
Source: PLoS One. 2013 Feb 11;8(2):e55904. doi: 10.1371/journal.pone.0055904 (PMC3569436; doi:10.1371/journal.pone.0055904)

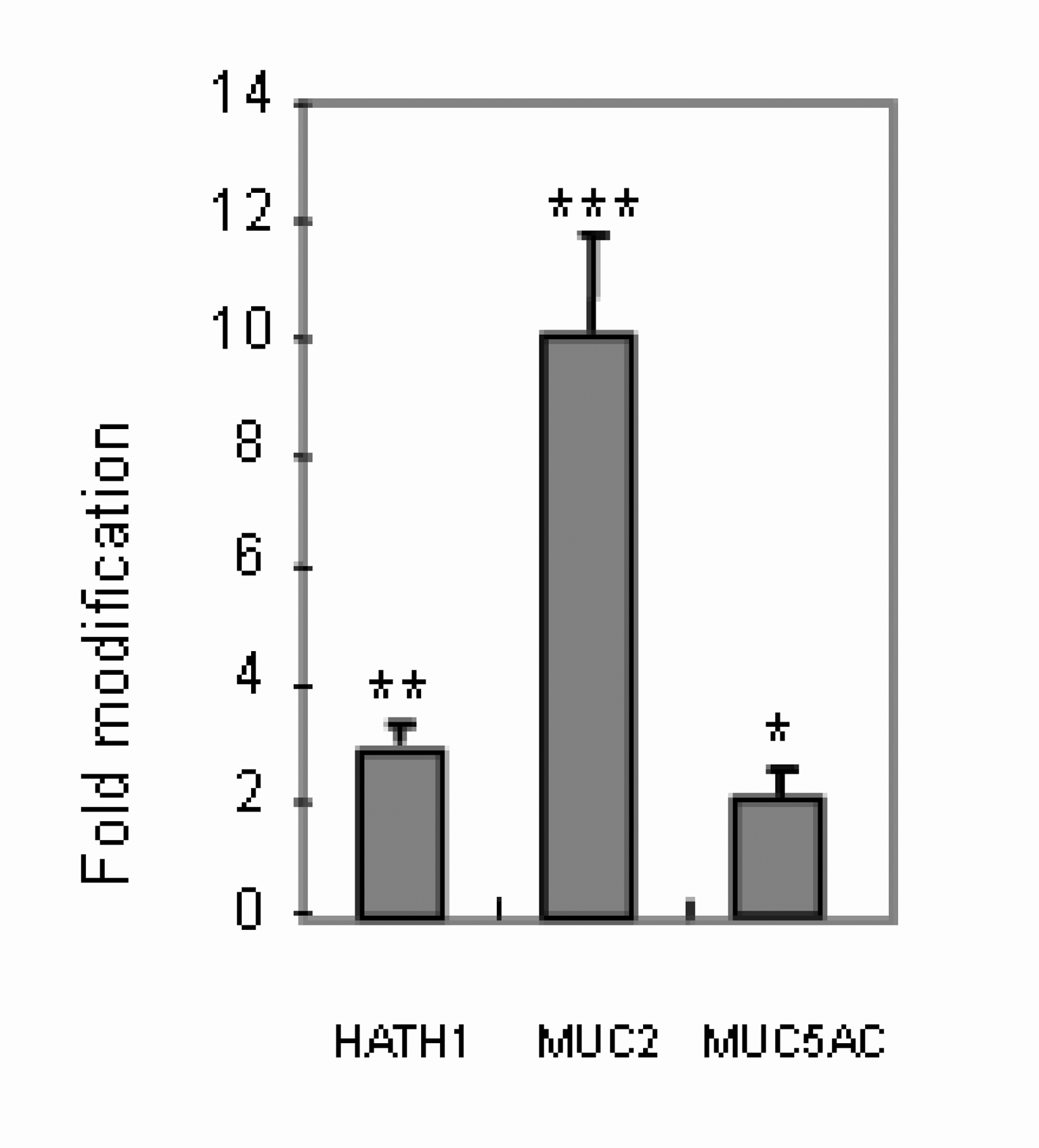

Supplement: Figure S1 — Effect of γ-secretase inhibition on differentiation parameters of HT29-Cl.16E-Sox9 cells. RT-PCR detection of Hath1, MUC2, MUC5AC mRNA in HT29-Cl.16E-Sox9 filter cultures treated with DBZ for 21 days. The results of real-time quantitative PCR are expressed relative to the expression level of control cultures after normalization to β-actin gene expression. Mean expression relative to control DMSO; Mean ± SE of 4 experiments; ***, P<0.001; **, P<0.01; *, P<0.05 (DBZ vs control). (TIF) [file pone.0055904.s001.tif]

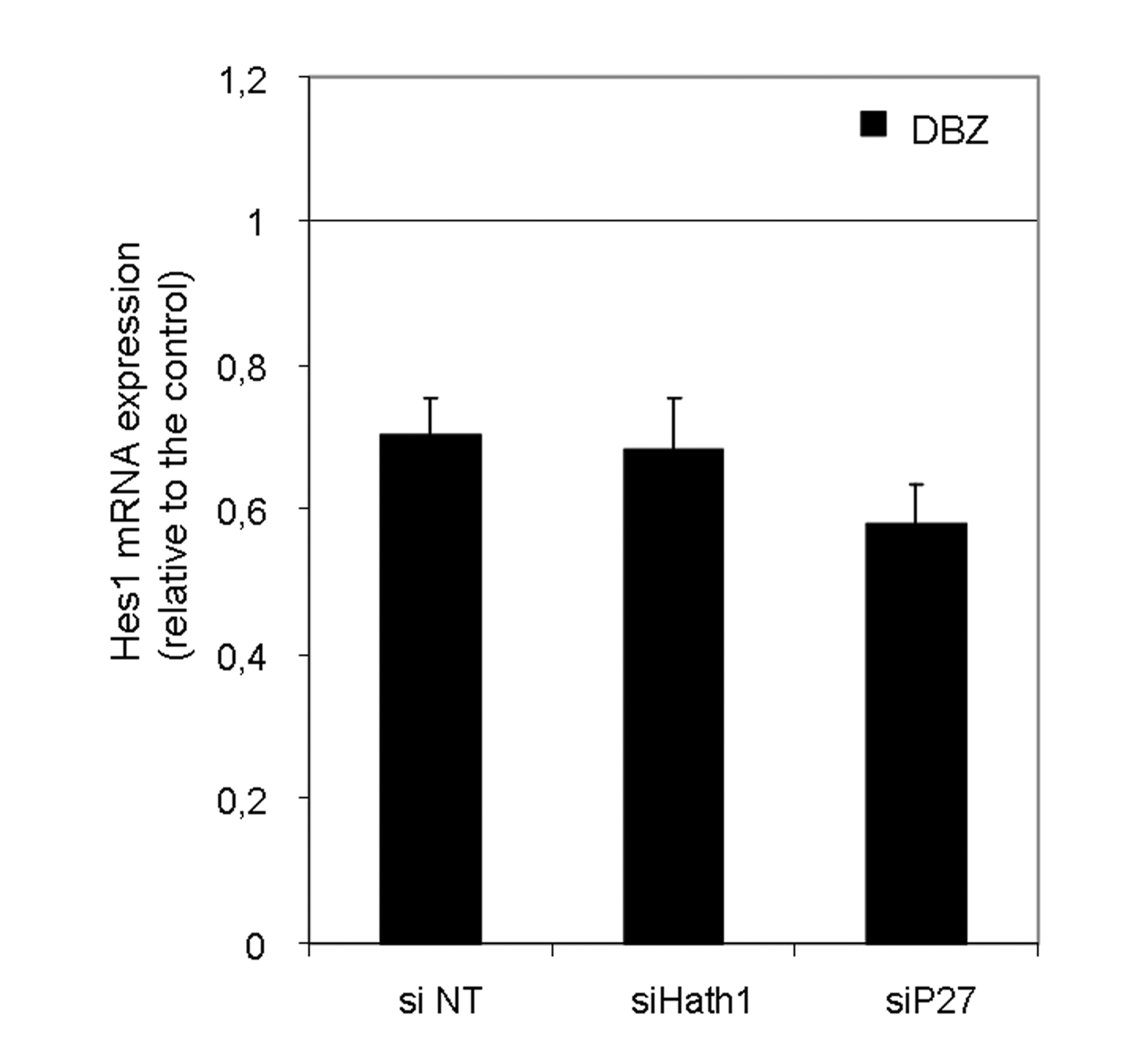

Supplement: Figure S2 — Effect of γ-secretase inhibition on Hes1 mRNA expression. Filter-grown HT29-Cl.27H, treated or not (control) with DBZ for 21 days, were resuspended and plated on polyHEMA-coated wells (nonadherent conditions) for 72 hours culture in the presence of the indicated siRNA target smart pool (NT, non target; Hath1 or P27Kip1). RT-PCR detection of Hes1 mRNA mean expression relative to siNT DMSO after normalization to actin gene expression; Mean ± SEM of 3 experiments. (TIF) [file pone.0055904.s002.tif]
